# Structure–Property Relationships of CO_2_ Absorbing Core–Shell Microparticles with Encapsulated Ionic Liquid

**DOI:** 10.1021/acsomega.3c02975

**Published:** 2023-06-17

**Authors:** Ai-Nhan Au-Duong, Asem Abdulahad

**Affiliations:** Department of Chemistry, Xavier University of Louisiana, New Orleans, Louisiana 70125-1056, United States

## Abstract

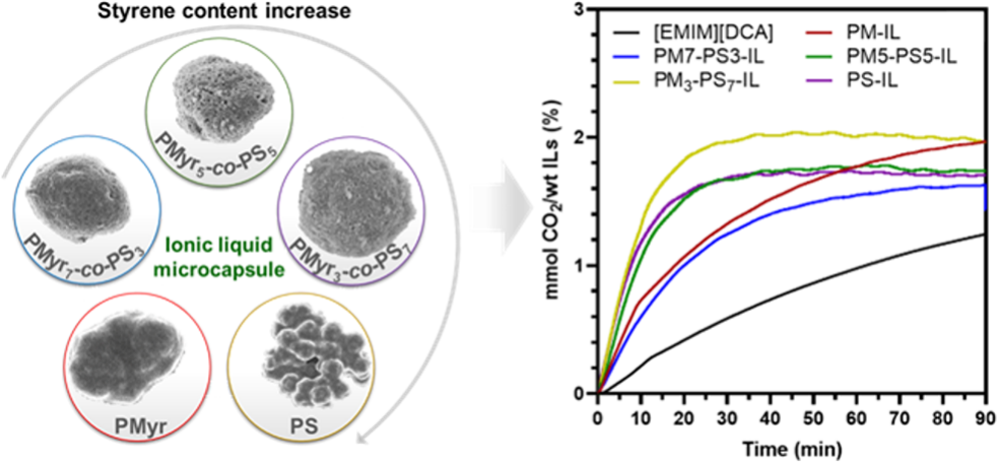

The demand for new ionic liquid (IL)-based systems to selectively sequester carbon dioxide from gas mixtures has prompted the development of individual components involving the tailored design of IL themselves or solid-supported materials that provide excellent gas permeability of the overall material as well as the ability to incorporate large amounts of ionic liquid. In this work, novel IL-encapsulated microparticles comprising a cross-linked copolymer shell of β-myrcene and styrene and a hydrophilic core of the ionic liquid 1-ethyl-3-methylimidazolium dicyanamide ([EMIM][DCA]) are proposed as viable materials for CO_2_ capture. Water-in-oil (w/o) emulsion polymerization of different mass ratios of β-myrcene to styrene (i.e. 100/0, 70/30, 50/50, 0/100) yielded IL-encapsulated microparticles, where the encapsulation efficiency of [EMIM][DCA] was dependent on the copolymer shell composition. Thermal analysis using thermogravimetric analysis (TGA) and differential scanning calorimetry (DSC) revealed that both thermal stability and glass transition temperatures depend on the mass ratio of β-myrcene to styrene. Images from scanning electron microscopy (SEM) and transmission electron microscopy (TEM) were used to observe the microparticle shell morphology as well as measure the particle size perimeter. Particle sizes were found to be between 5 and 44 μm. CO_2_ sorption experiments were conducted gravimetrically using TGA instrumentation. Interestingly, a trade-off between CO_2_ absorption capacity and ionic liquid encapsulation was observed. While increasing the β-myrcene content within the microparticle shell increases the amount of encapsulated [EMIM][DCA], the observed CO_2_ absorption capacity did not increase as expected due to reduced porosity compared to microparticles with higher styrene content in the microparticle shell. [EMIM][DCA] microcapsules with a 50/50 weight ratio of β-myrcene/styrene showed the best synergistic effect between spherical particle diameter (32.2 μm), pore size (0.75 μm), and high CO_2_ sorption capacity of ∼0.5 mmol CO_2_/g sample within a short absorption period of 20 min. Therefore, core–shell microcapsules composed of β-myrcene and styrene are envisioned as a promising material for CO_2_ sequestration applications.

## Introduction

Ionic liquids (ILs), a class of organic salts in the liquid state, have piqued extensive research interest in a wide range of academic and industrial fields, including catalysis, separation processes, analytics, lubricants, and various electrochemical applications.^1−5^ The molecular design of IL derived by appropriately selecting organic cations and anions is an important factor in designing IL solvents with specific properties and enabling high performance for various applications.^6^ Due to their excellent CO_2_ solubility and distinct physicochemical characteristics such as their negligible vapor pressure, high thermal and chemical stability, relative non-flammability, and design flexibility, these solvents have recently been researched for their potential application as alternative and sustainable absorption materials of greenhouse gases.^6−8^ Specifically for carbon dioxide sequestration, these properties make IL more favorable than conventional amine solvents that are corrosive and volatile because IL can be employed in harsher environments, and they mitigate the possibility of releasing sequestered CO_2_ back into the atmosphere.

Several efforts have been made to pursue IL as CO_2_ absorbents, with the general design principle based on structural modifications in the cation or anion with functional groups that are known to favorably capture CO_2_. For example, amino, carboxylate, alkoxide, phenolate, and azolate moieties are known to adsorb CO_2_.^9−14^ Despite their potential as CO_2_ absorbents, there are still many challenges that need to be addressed before IL can be widely employed in practical applications. For example, the high viscosity and surface tension of ionic liquids can result in slow mass transfer rates, making it difficult to incorporate them into existing processes and systems.^15,16^ Additionally, ionic liquids are often water-sensitive and expensive to produce and purify, which can limit their widespread adoption and implementation in industrial processes.^17,18^ Introducing IL onto solid supports could assist in retaining the active function of IL, improve mass transfer rates of CO_2_ into the IL, and increase the IL surface area available for CO_2_ sequestration. As a result, IL-based solids have the potential to improve IL performance, increase CO_2_ capture efficiency, and reduce costs.^15^

One strategy for introducing IL onto solid supports is through polymerization. For example, highly porous polymerized ionic liquids (PILs), IL-encapsulated microparticles, and cross-linked ion gels that incorporate anions such as bis(trifluoromethane) sulfonimide (Tf2N), trifluoromethanesulfonate (OTf), and dicyanamide (DCA) have been investigated for their ability to selectively sequester carbon dioxide from gas mixtures.^15,19−24^ Despite intensive research in this field, further research is needed to expand the view of material options and develop scalable and efficient methods for their use in CO_2_ capture.

Poly(β-myrcene) (PM) is a bio-based polyolefin material polymerized from myrcene (M), a renewable monomer found in plants such as conifers, wild thyme, hops, and bay leaves.^25−27^ The initial attraction of PM is its sustainable biomass availability and structural similarity to polyisoprene and polybutadiene. Generally, poly(β-myrcene) has a conjugated diene structure along with several interesting properties that include a relatively low glass transition temperature (*T*_g_, −68 °C), chemical resistance, and water resistance.^27^ Facilitating material design, a variety of polymerization methods, including living anionic polymerization, emulsion polymerization, and controlled and free radical polymerization, has significantly advanced the production of well-controlled PM homopolymers and copolymers.^27−29^ Hence, the synthetic utility of PM makes it an attractive option for its utilization as a solid support for IL. However, similar to natural rubbers, the hydrocarbon of PM is a non-porous, dense and continuous material in which pore development during polymerization is difficult for the homopolymer.^26^ To overcome this drawback, the incorporation of a comonomer is necessary to yield a network that results in porosity of rubbery PM-based materials and enable gas permeability.

**Scheme 1:**
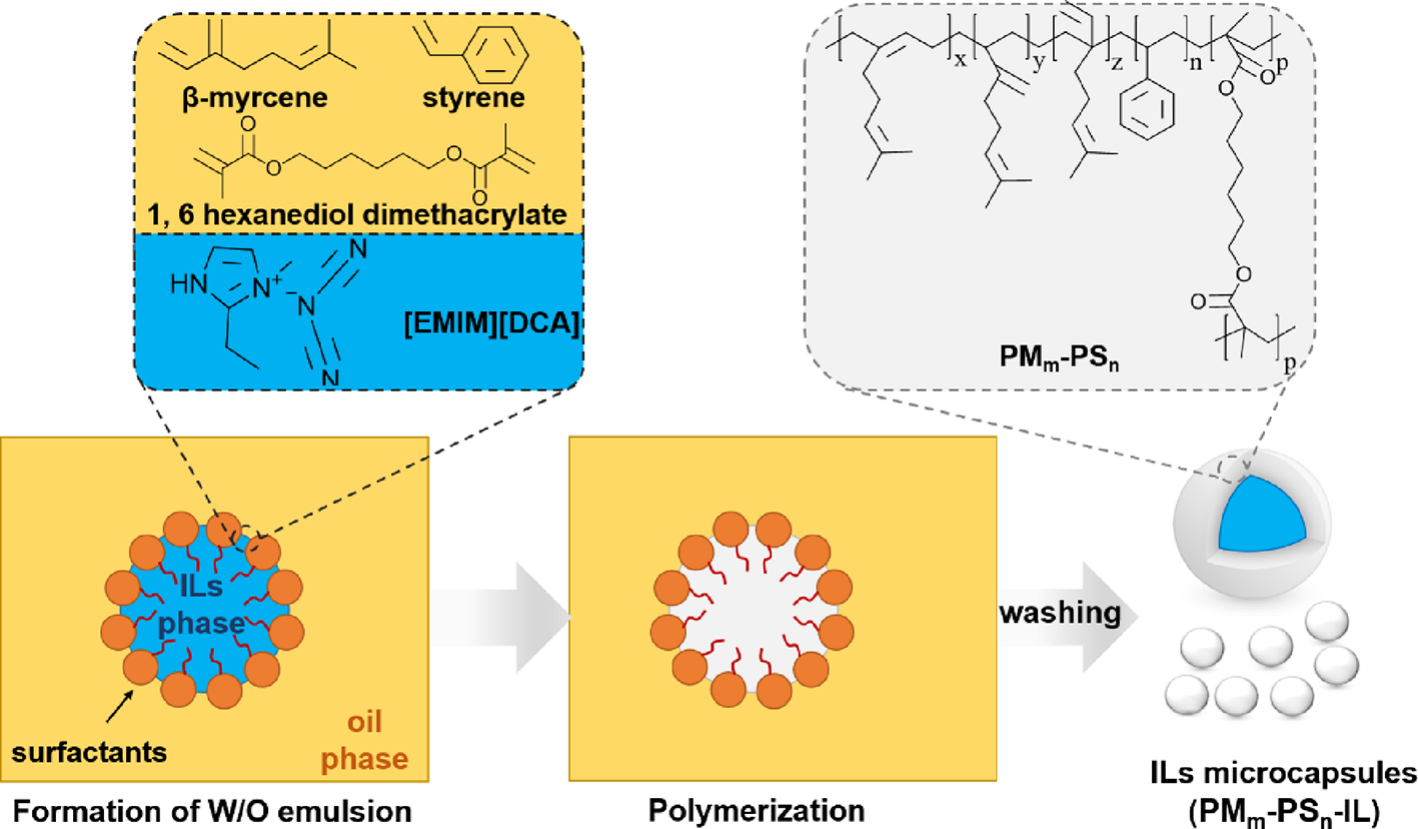
Preparation of PM_m_-PS_n_ Core–Shell Microparticles with a Copolymer Poly(β-myrcene)-Polystyrene Shell and an Ionic Liquid Core for Discretizing the Ionic Liquid into Micron-Size Drops to Increase Surface Contact Area and Mass Transfer Rate of CO_2_ into the Encapsulated IL

Herein, we report microcapsules comprising a cross-linked β-myrcene/styrene copolymer shell and [EMIM][DCA] core for discretizing the IL via micron-size particles resulting in increased porosity. Using emulsion polymerization, the pore size of [EMIM][DCA] encapsulating microparticles was found to depend on the molar ratio of myrcene-to-styrene monomers in the emulsion polymerization. Therefore, the particle surface contact area and, by extension, the mass transfer rate of CO_2_ to the encapsulated bulk IL is controlled through polymerization. Due to its large, rigid, and ring-shaped molecular phenyl (C_6_H_5_-) pendant, styrene has been used in various copolymer chains to unlock the porous structure of these systems by emulsion templating and emulsion polymerization methods.^30,31^ Additionally, the simultaneous microcapsule preparation via an emulsion polymerization technique should be available for large-scale processing, increasing the productivity of CO_2_-absorbing materials, and potentially allowing industrial-scale carbon capture.^32^ The water-in-oil emulsion was designed for the encapsulation of hydrophilic IL into a continuous phase containing a monomer mixture that facilitates the formation of a copolymer shell after polymerization (Scheme 1). Tween 20/Span 80 binary mixed surfactant systems with an eventual hydrophilic–lipophilic balance (HLB) of 6 was used to balance between the hydrophilic and hydrophobic portions of the mixture. 1-Ethyl-3-methylimidazolium dicyanamide ([EMIM][DCA]) was chosen as the model hydrophilic IL because of its inherent capability to adsorb CO_2_.^33,34^ A series of cross-linked PM_m_-PS_n_ microparticles with various PM/PS mole ratios were comprehensively studied for the effect of styrene content in the microcapsule shell on ionic liquid encapsulation, thermal properties, and microparticle morphology. Further, the ability for the IL-encapsulating PM_m_-PS_n_ microparticles to absorb CO_2_ was also evaluated.

## Experimental Section

### Materials

β-myrcene (stabilized, M), styrene (stabilized, S), 1-ethyl-3-methylimidazolium dicyanamide (≥98.0%, IL), Span 80, and Tween 20 were purchased from Sigma–Aldrich. 1,6-Hexanediol dimethacrylate (stabilized with MEHQ, HD) was purchased from TCI America. Ammonium persulfate (≥98.0%) and *n*-heptane were obtained from Acros Organics. β-Myrcene, styrene, and 1,6-hexanediol dimethacrylate were purified by passing through a column of aluminum oxide prior to use. All other chemicals were used as received.

### Synthesis of the PM_m_-PS_n_ Microparticles

[EMIM][DCA] encapsulation was accomplished by water-in-oil (w/o) emulsion polymerization. The aqueous dispersed phase included deionized water (1.0 g), ammonium persulfate (0.10 g), Tween 20 (0.056 g), and [EMIM][DCA] (1.2 g). The hydrophobic continuous phase was a mixture of heptane (14.0 g), Span 80 (0.4 g), and an appropriate mass of styrene and myrcene monomers as listed in Table S1. The aqueous and oil phases were prepared separately, mixed together, and then sonicated with a probe sonicator at 80% amplitude for 5 min. Prior to and throughout the sonication, the polymerization mixture was kept in an ice bath to prevent premature thermal initiation. The resulting stable w/o emulsion was degassed under nitrogen for 20 min before being heated to 70 °C with vigorous stirring for 24 h, except for the β-myrcene/styrene molar ratios of 30/70 and 0/100 with an 8 h reaction time. As the polymerization progressed, solid particles developed due to microparticle growth in the reaction medium. After the polymerization, the microparticles were collected by vacuum filtration and washed three times with heptane. Subsequently, microparticles were dried in a vacuum oven at 60 °C overnight and a yellow powder was obtained. It is also important to note that a hydrophilic–lipophilic balance (HLB) of 6 was used for the Tween 20/Span 80 binary surfactant mixture for all polymerizations. For comparison, IL-free microparticles were also prepared by the same procedure, where the [EMIM][DCA] ionic liquid was substituted with the same amount of DI water in the formula.

The ionic liquid content (i.e., encapsulation efficiency) within each sample was determined by measuring the mass difference before and after [EMIM][DCA] release using an acetone wash. Complete ionic liquid release from the encapsulating PM_m_-PS_n_ microparticles was confirmed using FTIR. To assess the mechanical integrity of the PM_m_-PS_n_ copolymer shell, the core–shell microparticles were redispersed in *n*-heptane and subjected to a series of centrifugation steps. Beginning with 3000 rpm, the speed of centrifugation was increased to 8000 rpm at a rate of 500 rpm/min. This test was done to determine if any [EMIM][DCA] would result from loss of shell integrity due to centrifugal force.

### Instrumental Methods

Fourier transform infrared spectroscopy–attenuated total reflectance (FTIR-ATR, Nicolet 380) was used for chemical characterization of the prepared microparticles and the bulk ionic liquid. For each sample, 32 scans were accumulated within a spectral range of 4000–400 cm^–1^ at 25 °C.

Thermogravimetric analysis (TGA) was accomplished using a TA instruments TGA 5500 equipped with an autosampler. Under a nitrogen atmosphere, each sample was heated to 100 °C for 60 min to ensure that trace solvents were removed prior to TGA measurement. The sample was then cooled, equilibrated at 50 °C, and heated to 600 °C at a heating rate of 10 °C/min. Differential scanning calorimetry (DSC) measurements were completed using a TA Instruments DSC 2500 under a nitrogen atmosphere using a heat/cool/heat protocol where the sample was heated from −80 to 120 °C at a rate of 10 °C/min and cooled back to −80 °C at a rate of 5 °C/min before reheating to 120 °C. Between heating and cooling cycles, the sample was equilibrated for 10 min.

Scanning electron microscopy (SEM) images were obtained from a Hitachi S-4800 FESEM at an accelerating voltage of 5 kV. For SEM images, the solid polymer microparticles were dispersed in *n*-heptane, cast onto a glass cover slide, and allowed to dry. After drying the sample was sputter-coated with gold at 2.0 mA for 1 min using a Quorum Technologies K550X sputter coater. Transmission electron microscopy (TEM) images were obtained using a Hitachi HT7700. TEM samples were prepared by redispersing polymerized microparticles into heptane and dispersing onto a copper TEM grid. ImageJ software was used to process and analyze the images.

### Carbon Dioxide Absorption Capacity

CO_2_-absorption capacity was determined gravimetrically using TGA instrumentation. Samples were heated to 100 °C for 60 min to remove any residual solvent or moisture under a nitrogen atmosphere. Subsequently, the sample was cooled to 25 °C and the TGA gas was switched to carbon dioxide. The core–shell microparticles were exposed to the carbon dioxide atmosphere for 60–90 min, and the mass of CO_2_ uptake was measured throughout.

## Results and DISCUSION

### Preparation and Characterization of IL-Encapsulated Microparticles

Water-in-oil (w/o) emulsion polymerization was used to encapsulate [EMIM][DCA] ionic liquid within a cross-linked, hydrophobic shell comprised of β-myrcene, styrene, or copolymerized β-myrcene and styrene monomers. [EMIM][DCA] was used as the ionic liquid core because of its well-known ability to adsorb CO_2_ via interaction between cyano functional groups in the dicyanamide anion and the electron deficient carbon atom in CO_2_.^33,34^ Using a binary surfactant system of Tween 20 and Span 80 (HLB = 6) to stabilize the emulsion, [EMIM][DCA], ammonium persulfate, and water were dispersed throughout the hydrophobic continuous phase using probe sonication. Employing ammonium persulfate as the water-soluble initiator facilitated thermal initiation and polymerization at the water–oil interface to produce core–shell microparticles. The hydrophobic phase included the monomers comprising the microparticle shell (i.e. styrene, β-myrcene, or both) and the 1,6-hexanediol dimethacrylate cross-linker, where the cross-linker was held constant for all polymerizations. β-Myrcene/styrene molar ratios of 100/0, 70/30, 50/50, 30/70, and 0/100 were used in the monomer feedstock to prepare samples with varying chemical composition in the copolymer shell of the microcapsules. Core–shell microparticles were also polymerized in the absence of [EMIM][DCA] (denoted as IL-free). IL-free particles were prepared using the same conditions except the [EMIM][DCA] was replaced by an equivalent amount of deionized water. The samples collected were named PM, PM_7_-PS_3_, PM_5_-PS_5_, PM_3_-PS_7_, and PS to represent the myrcene/styrene molar ratios of 100/0, 70/30, 50/50, and 0/100, respectively.

**Figure 1:**
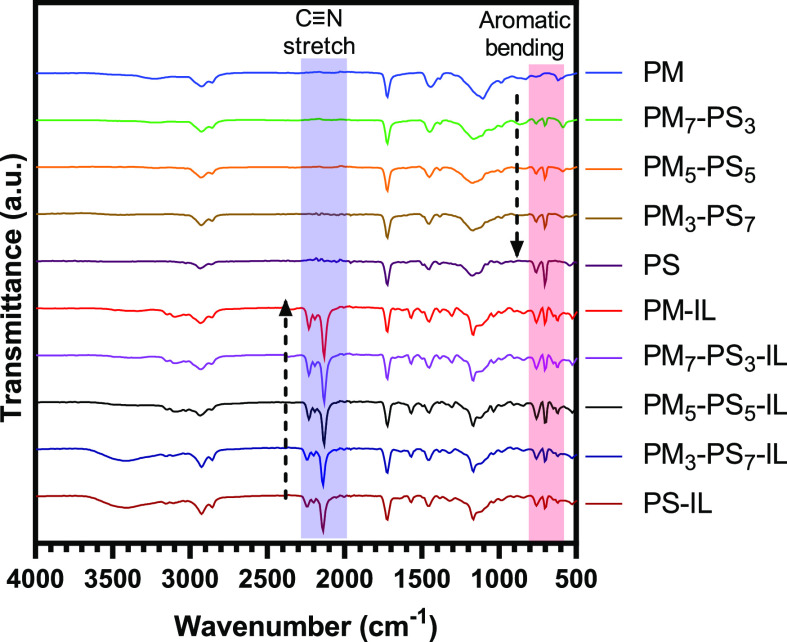
ATR-FTIR spectra of ionic liquid containing PM, PS, PM_m_-PS_n_ core–shell microparticles with encapsulated [EMIM][DCA] and ionic liquid-free PM_m_-PS_n_ microparticles.

Figure 1 and supplemental Figure S1 show FTIR-ATR spectra of the neat [EMIM][DCA] ionic liquid, PM, ionic liquid containing PM_m_-PS_n_ core–shell microparticles, and ionic liquid free PM_m_-PS_n_ microparticles. Broad absorption peaks in the region of 2850 and 2925 cm^–1^ were identified as the stretching vibration of −CH and −CH_2_ groups on both styrene and myrcene. The absorption peak located at 3025 cm^–1^ was identified as the stretching vibration of −CH_3_ groups from myrcene. For the sample of cross-linked PS particles, the characteristic peaks from monosubstituted aromatic sp^2^-hybridized C–H bending was observed at 700 and 755 cm^–1^. In contrast, the cross-linked PM sample did not show any peaks at these positions because there are no aromatic C–H groups in the β-myrcene structure. Consequently, the monosubstituted aromatic sp^2^-hybridized C–H bending frequencies are also observed in the FTIR spectra of PM_m_-PS_n_ copolymerized core–shell microparticles, which indicates the successful inclusion of styrene into the structure of the myrcene-based microparticles. All the samples displayed a high intensity absorption band from the carbonyl vibration (1725 cm^–1^) that is contributed by the 1,6-hexanediol dimethacrylate cross-linker used in the polymerization. It is important to note that the amount of 1,6-hexanediol dimethacrylate was held constant for all polymerizations. Therefore, the carbonyl absorption peak observed at 1725 cm^–1^ may be used as a reference (i.e., internal standard) for comparing the intensity of other FTIR absorption bands to estimate the mole percent of styrene within the microparticle shell.

The intensity of the aromatic C–H bending peak gradually increases with increasing styrene content in cross-linked PM_m_-PS_n_ microparticles. The mole fraction of styrene in each copolymer microparticle was calculated by determining the ratio of the intensity of the aromatic C–H peak at 700 cm^–1^ to the carbonyl peak at 1725 cm^–1^ and comparing the ratios to the same ratio determined from the pure polystyrene microparticles. Based on this analysis, the PM_7_-PS_3_, PM_5_-PS_5_, and PM_3_-PS_7_ microparticles that were prepared using molar feed stock ratios of 7/3, 5/5, and 3/7 (β-myrcene/styrene) yielded copolymerized microparticles with styrene mole fractions of 0.23, 0.49, and 0.58, respectively.

**Figure 2:**
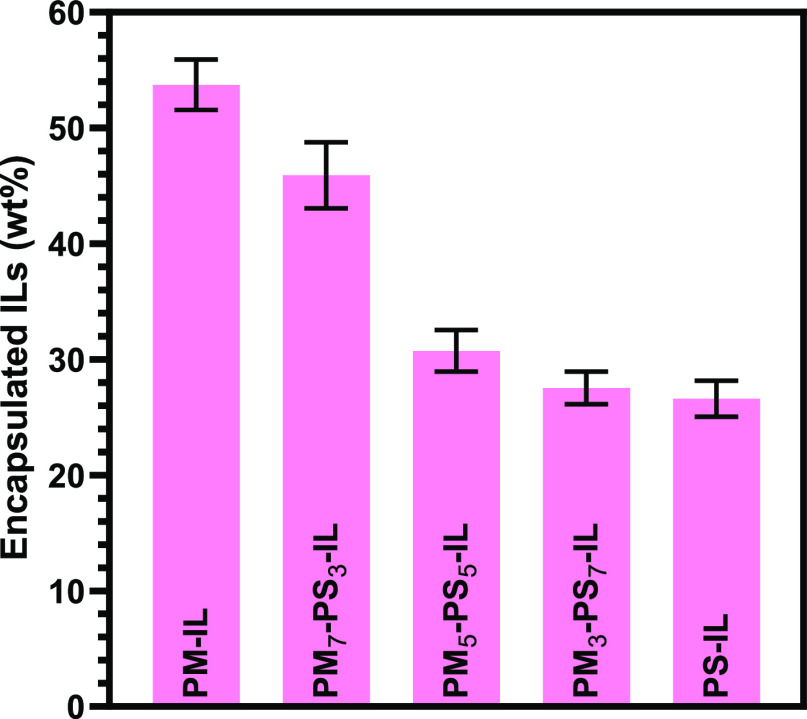
wt % of encapsulated [EMIM][DCA] within PS, PM, and PM_m_-PS_n_ core–shell microcapsules as determined by measuring weight difference before and after ionic liquid removal using a serial acetone wash.

Comparing the representative spectra for both the neat and encapsulated [EMIM][DCA] spectra shown in Figure 1, an antisymmetric C≡N stretching vibration at 2130 cm^–1^ is attributed to the dicyanamide anion in the ionic liquid structure. The C≡N stretch is present in ionic liquid-encapsulating samples and absent in the FTIR spectra of the IL-free samples, which confirms the presence of [EMIM][DCA] in the ionic liquid encapsulated samples. FTIR spectra of the IL-encapsulated samples show that the antisymmetric C≡N stretching vibrations were found to decrease steadily at higher styrene content in the copolymer microparticles. This suggests that the amount of [EMIM][DCA] decreases with increasing styrene content in the PM_m_-PS_n_ microparticle shell. To confirm this observed trend in the FTIR spectra, the encapsulation efficiency of each PM_m_-PS_n_ particle was determined experimentally from the mass recovery of the ionic liquid after thoroughly washing the microparticles with acetone comparing the mass difference before and after release of [EMIM][DCA]. Complete removal of the ionic liquid from each sample was confirmed by FTIR (Figure S2). The percentage of encapsulated ionic liquid (depicted in Figure 2 and listed in Table 1) was determined to be 54, 46, 31, 28, and 26 wt % for PM, PM_7_-PS_3_, PM_5_-PS_5_, PM_3_-PS_7_, and PS, respectively. Overall, FTIR analysis of PM_m_-PS_n_ particle with and without encapsulated [EMIM][DCA] shows that the w/o emulsion polymerization using a binary surfactant system of Tween 20 and Span 80 yielded sufficient control over the chemical composition of copolymerized microparticle shells and that the encapsulation efficiency of the [EMIM][DCA] ionic liquid decreased with increasing styrene content in the microparticle shell.

**Table 1:** List of the Composition and Thermal and Physical Properties of PM_m_-PS_n_ Microparticles

sample	styrene mole fraction^a^ (*I*_700_/*I*_1725_)	[EMIM][DCA] content^b^ (wt %)	*T_d,5%_* (°C)^c^	*T*_g_ (°C)^d^	average particle size^e^ (μm)	average pore size^e^ (μm)
PM			215	18		
PM-IL		54	264	16	23.9 ± 4.9	
PM_7_-PS_3_	0.23		217	43		
PM_7_-PS_3_-IL		46	264	49	35.4 ± 4.4	0.74 ± 0.08
PM_5_-PS_5_	0.49		247	46		
PM_5_-PS_5_-IL		31	259	52	32.2 ± 3.8	0.75 ± 0.1
PM_3_-PS_7_	0.58		257	61		
PM_3_-PS_7_-IL		28	264		44.1 ± 17.3	0.5 ± 0.05
PS	1.00		252			
PS-IL		26	265		4.5 ± 0.9	

a Calculated from FTIR spectra using the intensity of the carbonyl stretch at 1725 cm^–1^ as an internal standard.

b Determined experimentally after removing the [EMIM][DCA] ionic liquid by washing with acetone.

c Determined by thermogravimetric analysis.

d Determined by differential scanning calorimetry.

e Measured by analysis of SEM images using ImageJ software.

### Thermal Analysis of PM_m_-PS_n_ Microparticles

**Figure 3:**
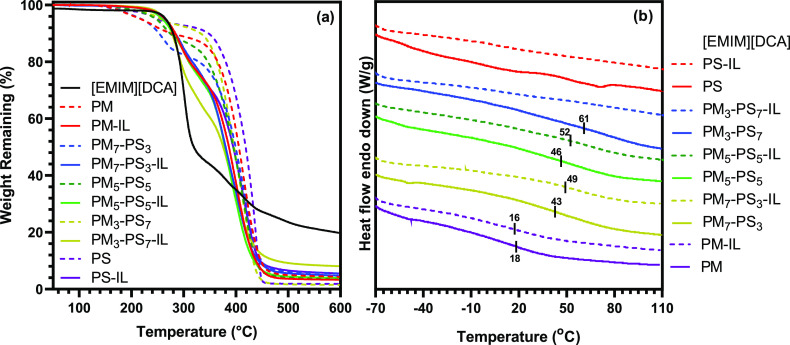
Thermal characterization of IL-free and IL-encapsulated PM_m_-PS_n_ by (a) thermogravimetric analysis and (b) differential scanning calorimetry.

Figure 3 shows the results of thermal characterization of PM_m_-PS_n_ microparticles using thermogravimetric analysis (TGA) and differential scanning calorimetry (DSC). The degradation temperatures at 5% mass loss, *T*_d,5%_, and the glass transition temperatures, *T*_g_, for each sample are listed in Table 1. As shown in Figure 3a, TGA curves for PM_m_-PS_n_ microparticles show a two-stage degradation where the first stage occurs between 200 and 350 °C and the second occurs from 350 to 455 °C for all microparticles. With *T*_d,5%_ > 200 °C for all PM_m_-PS_n_ microparticles, the thermal stability increases slightly with an increase in styrene content of the microparticle shell. It is important to note that pure polystyrene typically shows a single-step thermal degradation by TGA.^35^ Therefore, the first stage of thermal degradation is attributed to the degradation of ester functional groups contributed by the 1,6-hexanediol dimethacrylate cross-linker and may also include degradation of residual unreacted β-myrcene monomers. Also, the higher thermal stability of polystyrene compared to poly(β-myrcene) was demonstrated by the degradation patterns of the two cross-linked homopolymers (5% mass loss after 215 °C for PM and after 252 °C for PS). This increased thermal stability is ascribed to incorporation of the benzene side group from styrene within the cross-linked shell structure. The degradation behavior of IL-encapsulating PM_m_-PS_n_ core–shell microparticles similarly exhibits higher degradation temperatures. Since the neat IL has higher degradation temperature *T*_d,5%_ at 265 °C, the presence of IL in their structure leads to a significant impact on thermal degradation behavior of the microparticles. Therefore, the thermal degradation of PM_m_-PS_n_ core–shell microparticles depends on both the copolymer composition of the shell and the encapsulated ionic liquid.

The impact of copolymer composition on the thermal properties of PM_m_-PS_n_ is also observed in their glass transition temperatures as determined by DSC (shown in Figure 3b and listed in Table 1). First, it is noteworthy that the DSC traces show broad glass transitions, which are indicative of cross-linked materials. The DSC traces in Figure 3a reveal an amorphous structure with a shift in glass transition temperatures toward a higher temperature as β-myrcene content was reduced in the copolymer microparticle shells. Considering the structure of pure poly(β-myrcene), which consists of flexible chains resulting in a rubbery material with low *T*_g_, decreasing the β-myrcene content in the PM_m_-PS_n_ copolymer microparticles increases macromolecular chain stiffness, which results in higher *T*_g_ due to higher percent incorporation of styrene within the cross-linked structure.^36^ Different from the inclusion of styrene, comparing the IL-free to the IL-encapsulated microparticles revealed that the [EMIIM][DCA] encapsulation did not show a significant influence over the *T*_g_, which suggests good chemical compatibility between the encapsulated ionic liquid and the cross-linked copolymer shell. In addition, the DSC trace for the pure [EMIM][DCA] that is shown in the Supplemental Information (Figure S3) shows clear crystallization and melting transitions for the ionic liquid. These transitions were not observed for the ionic liquid encapsulated PM_m_-PS_n_ core–shell microparticles, which suggests successful discretization of the [EMIM][DCA] into micron-sized droplets within the microcapsule core.

### Morphology

**Figure 4:**
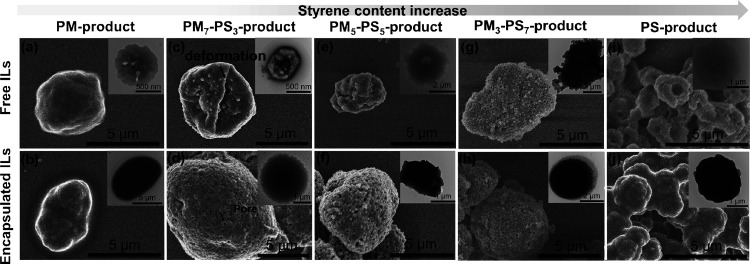
FE-SEM images for (a) PM, (b) PM-IL, (c) PM_7_-PS_3_, (d) PM_7_-PS_3_-IL, (e) PM_5_-PS_5_, (f) PM_5_-PS_5_-IL, (g) PM_3_-PS_7_, (h) PM_3_-PS_7_-IL, (i) PS, and (j) PS-IL. The inset images show TEM images of the corresponding sample.

SEM and TEM were used to image oven-dried powder samples of aggregated microparticles to investigate the effect of β-myrcene/styrene composition and IL content on the morphology of the PM_m_-PS_n_ core–shell microparticles. Figure 4 and Figure S4 show the SEM and TEM images of the five different microparticle sample sets (i.e., PM, PS, and PM_m_-PS_n_ microparticles with and without encapsulated ionic liquid). In addition, the average particle size of PM_m_-PS_n_ (listed in Table 1) is the average perimeter of the microparticles, where the average perimeter was between 32 and 44 μm for the copolymer microcapsules. The average sizes of 24 and 5 μm for the homopolymer PM and PS microparticles, respectively, were significantly diminished compared to that of the PM_m_-PS_n_ copolymeric microparticles. Considering the removal of residual solvent and water during the drying step used to prepare the TEM and SEM samples, microparticle shrinkage from the formation of a cavity within the core was anticipated. Microparticles that were polymerized in the absence of ionic liquid are shown in the top row of images in Figure 4a,c,e,g,i. After drying to prepare the samples for imaging, the IL-free samples appear flat and misshapen, which confirms that there was empty space within the core after removing water from the microparticles. Microparticles with encapsulated IL are shown in the bottom row of images in Figure 4b,d,f,h,j. In contrast to the IL-free microparticles, microparticles with encapsulated [EMIM][DCA] did not fully deflate after drying and appear to have maintained their general spherical/ellipsoid shape. Accounting for the misshapen ellipsoids of each microparticle, removing solvent from the core of the IL-free particles should create a void within the core, and the shell of swollen microparticles would deform slightly to lose its general ellipsoid shape.

Field emission TEM images (the inset images within Figure 4) supports the FTIR chemical analysis by showing that the IL-encapsulated microparticles possess a distinct core–shell structure compared to the IL-free particles. This observation is based on the darker contrast surrounded by a lighter gray-tone shell observed in the TEM images taken from particles with encapsulated [EMIM][DCA], which suggests that the encapsulated ionic liquid is the darker contrast, and the light gray-tone shell is the polymeric shell. The polymer shell of the IL encapsulating microparticles also provides sufficient protection from IL leaching. This was demonstrated by the lack of free ionic liquid after subjecting the IL-encapsulated microparticle suspensions in *n*-heptane to high centrifugal force from 3000 to 8000 rpm and a sequential increment of 500 rpm per minute for each centrifuging step (Figure S5). Conversely, the IL-free cross-linked particles, shown in Figure 4a,c,e,g,i, appeared as a uniform region of bright gray color.

Additionally, visualizing the morphology using SEM and TEM, revealed significant variation in the appearance of the microparticles’ surface, which provides further insight into the polymerization of the polymeric shell at the interface between the dispersed aqueous phase and the hydrophobic continuous phase. Specifically, the inclusion of styrene units in the cross-linked copolymer shell was found to influence the polymer shell surface morphology. Comparing the SEM images in Figure 4b,j, the surfaces of the homopolymer PM and PS core–shell microparticles appear smooth with no readily apparent pores. However, the pure PS particles (Figure 4j) were especially diminished in size and were observed as an aggregated array of conjoined particles. The decreased size was attributed to a larger central cavity resulting from sample drying during SEM/TEM sample preparation, where the PS particles exhibited greater shrinkage and deformation. It is noteworthy that styrene monomers have higher water solubility than β-myrcene monomers (300 vs 4.09 mg/L at 25 °C) due to its increased polarity.^37^ Based on its higher affinity for water compared to β-myrcene, styrene monomers are more likely to diffuse from the hydrophobic continuous phase into the dispersed aqueous phase during the polymerization, where the water-soluble initiator is available to initiate the polymerization. Therefore, we hypothesize that the transport of emulsified monomer reservoirs into the dispersed aqueous phase led to self-assembly deeper within the dispersed aqueous droplets after preparing the emulsion and that this phenomenon accounts for both the diminished size of the microparticles as well as the increased deformation. Additionally, in the presence of the 1,6-hexanediol dimethacrylate cross-linker, the individually small PS spheres may append and form particle arrays surrounding the large aqueous reservoir, which was removed after washing and drying steps, resulting in a larger central cavity for microparticles containing higher styrene monomer concentration in prepared emulsions. In the case of microparticles with high β-myrcene content, the polymerization preferentially occurs at the water/oil interface due to the increased hydrophobicity of β-myrcene compared to styrene. Additionally, when styrene is absent from the monomer feedstock, emulsion polymerization of PM yields a dense outer layer with a larger inner core where increased amounts of [EMIM][DCA] can reside.

For copolymerized PM_m_-PS_n_ microcapsules when β-myrcene concentration is high, the incorporation of styrene into the growing β-myrcene chains likely prevents the hydrocarbon polymer chains from closely packing by interrupting intermolecular interactions between the chains to allow the chains to self-assemble into porous structures.^30,31^ This is most apparent in the copolymerized IL-encapsulating PM_m_-PS_n_ microparticles shown in Figures 4d,f,h, where SEM imaging clearly shows an increase in microparticle shell porosity with increased styrene content. More specifically, the porous structure was observed when the styrene monomer content in the copolymer molar feedstock ratio increased from 30% to 70% molar. The pore sizes as measured by ImageJ software are listed in Table 1. The average pore sizes were measured to be 0.74–0.75 μm for PM_m_-PS_n_ particles produced from β-myrcene/styrene ratios of 70/30 and 50/50 and were slightly reduced to 0.50 μm for the 30/70 β-myrcene/styrene ratio. Based on images from TEM and SEM, the morphology is consistent with the IL encapsulation efficiency studies (Figure 2), which revealed that higher styrene content in the microparticle shell leads to reduced IL encapsulation. Based on the influence of styrene during the emulsion polymerization leading to smaller particle sizes and larger void formation upon drying, we posit that, during particle formation, [EMIM][DCA] was likely lost during the core–shell microparticle formation.

### CO_2_ Sorption and Selectivity

**Figure 5:**
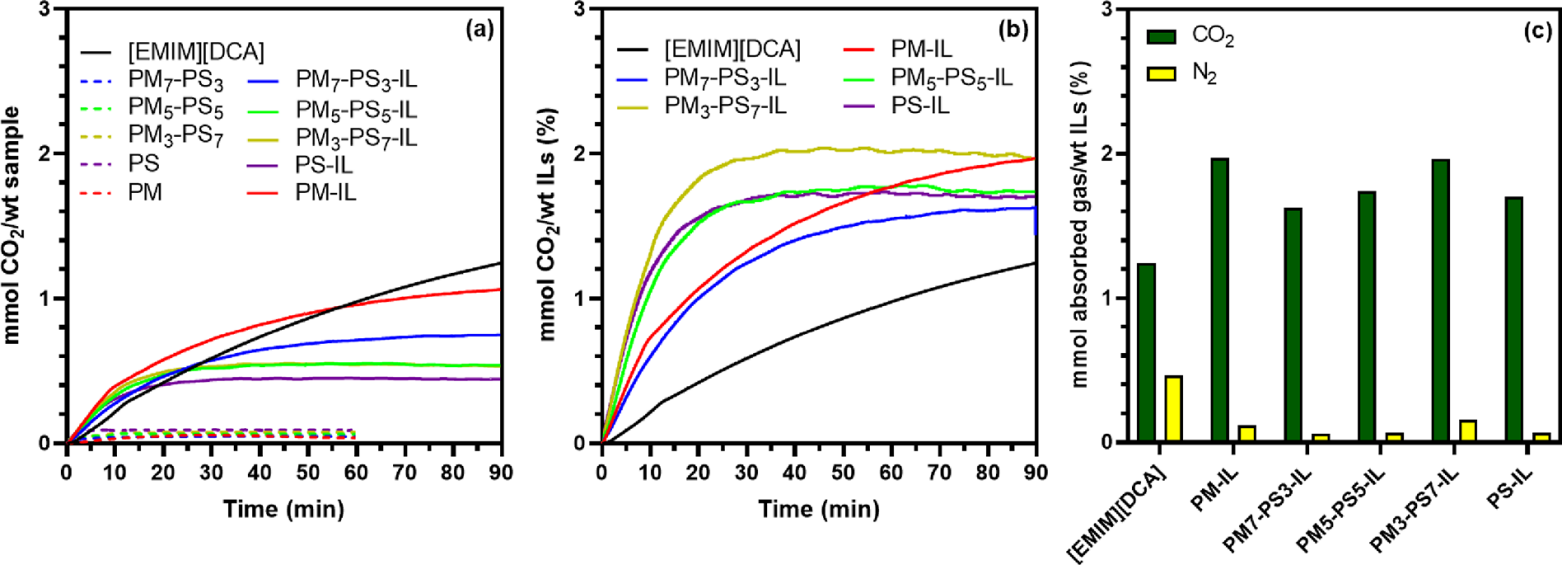
Carbon dioxide absorption of IL-free and IL-encapsulated microparticles portrayed per (a) mass of prepared samples and (b) mass of [EMIM][DCA], and (c) bar graph comparing CO_2_ and N_2_ sorption of each sample.

Ionic liquid-encapsulating PM_m_-PS_n_ microparticles were also evaluated for their ability to capture CO_2_. To identify the influence of IL content, particle size, and shell morphology on gas sorption behavior, an absorption experiment was conducted gravimetrically at a constant temperature of 25 °C and atmospheric pressure with a CO_2_ flow rate of 10 mL/min. Absorption curves showing CO_2_ uptake over time are presented in Figure 5. From the plot of mmol CO_2_ absorbed per overall mass of the sample in Figure 5a, the IL-free microparticles did not show any apparent ability to absorb CO_2_ over 60 min. Conversely, the [EMIM][DCA]-encapsulating core–shell microparticles showed a significant ability to absorb CO_2_ throughout 90 min of exposure to CO_2_ gas. This is due to the ability of the dicyanamide anion that is contributed by the [EMIM][DCA] ionic liquid to bind CO_2_. Based on the density functional theory (DFT) calculations published by Bhargava et al.,^38^ the electron deficient carbon atom in the CO_2_ molecule is able to interact with the negatively charged dicyanamide anion in a manner that resembles a Lewis acid–base interaction. As anticipated, Figure 5a also shows that the CO_2_ absorption capacity increases with increasing mass of IL; bulk [EMIM][DCA] absorbs more CO_2_ over time and core–shell particles with less styrene content have a higher absorption capacity because more ionic liquid is present when styrene content is lower. As discussed previously (Figures 2 and 4 and listed in Table 1), the decrease in encapsulation efficiency of [EMIM][DCA] by the core–shell PM_m_-PS_n_ microparticles is likely due to smaller particles sizes and larger void formation as the styrene content increases. Despite the relative similarity in overall absorption capacity across the samples, when styrene content is higher in the microcapsule shell, there is less ionic liquid available for CO_2_ sorption, which results in lower CO_2_ absorption capacity. Hence, increasing the styrene content of the copolymer microparticle shell enhances the porosity of the shell but decreases the total amount of encapsulated ionic liquid.

CO_2_ sorption experiments also revealed that the copolymer microcapsules demonstrated better overall absorption behavior and faster kinetics. Figure 5b shows the amount of CO_2_ absorbed per unit mass of ionic liquid. When accounting only for the mass of ionic liquid, the absorption of CO_2_ by the IL-encapsulated microcapsules proceeded faster with shorter saturation time than that of liquid IL. The CO_2_ saturation absorption times of IL-encapsulated PM_5_-PS_5_, PM_3_-PS_7_, and PS microparticles were achieved after approximately 20 min, whereas the PM_7_-PS_3_ required more than 60 min to equilibrate. For the PM-IL and neat IL, the CO_2_ absorption results similarly show longer saturation times of >90 min. These results are attributed to an increase in available IL surface contact area via discretizing the IL after the incorporation of [EMIM][DCA] into the core–shell microcapsules and an increase in shell porosity with increased styrene content. These results also suggest that IL-encapsulation reduces the impact of high resistance to mass transfer in ionic liquids,^15^ and their low carbon dioxide diffusion coefficients.^39^ Interestingly, the nonporous PS-IL microparticles have a roughly similar mass transfer rate to the PM_5_-PS_5_ and PM_3_-PS_7_ copolymer core–shell microparticles. Presumably, this is due to two factors: (1) PS-IL particles have greater surface area that results from their significantly smaller average particle size compared to the copolymer core–shell particles, and (2) the PS homopolymer shell is thinner compared to the copolymer PM_m_-PS_n_ copolymer shells. Based on our analysis, the IL microcapsule samples with 50/50 ratio of β-myrcene/styrene outperformed the other prepared particles when considering the ionic liquid content (31 wt %), spherical particle formation (perimeter of 32.2 μm), average pore size of 0.75 μm, high CO_2_ absorption capacity of approximately 0.5 mmol CO_2_/g sample, and fast mass transfer (saturation reached after 20 min).

Comparing the 50/50 microparticle sample to the 70/30 microparticle sample, both had similar average particle size and similar average pore size. Among the porous copolymer microparticles, both of these samples combined small particle size and large pore size, which are both advantageous for high mass transfer rate of CO_2_ into the microparticles. However, the 50/50 microparticle sample showed improved CO_2_ absorption capacity and faster CO_2_ saturation (Figure 5a,b). Comparing the CO_2_ absorption performance of the 50/50 and 30/70 microparticles, the 50/50 microparticle samples absorb more CO_2_ overall per mass of the sample despite having slower CO_2_ saturation and lower CO_2_ absorption per mass of encapsulated ionic liquid. This observation is most likely explained by the discretization of the ionic liquid within the smaller microparticles that have a 50/50 β-myrcene/styrene shell composition. Further, the copolymerized PM_5_-PS_5_ microparticles incorporate a higher percentage of β-myrcene monomer compared to the PM_3_-PS_7_ microparticle sample, which enables the use of renewable and sustainable β-myrcene in CO_2_ sequestration applications.

To demonstrate their selectivity, PM_m_-PS_n_ microparticles were also exposed to N_2_ gas using the same experimental parameters as the CO_2_ sorption experiments. Nitrogen absorption isotherms are shown in Figure S6, and Figure 5c shows a comparison of the CO_2_ and N_2_ absorption capacity. Nitrogen absorption experiments show a nearly negligible uptake of N_2_ by all samples. This result is consistent with high selectivity of [EMIM][DCA] described in the literature.^34^ Overall, this work presents a novel material for CO_2_ capture using ionic liquid encapsulated PM_m_-PS_n_ cross-linked copolymer core–shell microparticles. These copolymer microparticles show a promising ability to both protect the [EMIM][DCA] ionic liquid and boost the mass transfer of CO_2_ into the microcapsule while maintaining the high selectivity and affinity of the ionic liquid for CO_2_ compared to N_2_.

Overall, this work shows the potential of emulsion polymerization as a means to synthetically control the encapsulation of a CO_2_ absorbing ionic liquid within core–shell microparticles equipped with a porous polymer shell. Based on their fast CO_2_ absorption, their competitive CO_2_ absorption capacity, and their selectivity for CO_2_ over N_2_, these materials can be envisioned as a component polymer-based membranes for use in post-combustion CO_2_ capture systems.^40^ As a dispersed component within a polymer matrix, the ability to encapsulate [EMIM][DCA] increases the effective surface area for CO_2_ sequestration via discretization of the ionic liquid, and the use of readily available styrene and sustainable β-myrcene reduces the cost of production. In addition, by adjusting the HLB or type of emulsifier employed, the cosurfactant strategy for accomplishing water-in-oil emulsion polymerization to produce core–shell microparticles can be adapted to encapsulate any aqueous soluble species with an affinity for CO_2_ that may have higher CO_2_ absorption capacity and increased selectivity.

## Conclusions

Novel IL-encapsulating core–shell microparticles were fabricated using w/o emulsion polymerization. Control over the β-myrcene/styrene molar feedstock ratio yielded several microcapsules with varying chemical composition in the microparticle shell which, in turn, resulted in varying amounts of [EMIM][DCA] ionic liquid encapsulated within the microparticle core. The morphology, particle size, and pore sizes on the surface of the microcapsules were also observed to depend on the monomer feedstock composition. All prepared microcapsules exhibited relatively high thermal stability (*T*_d,5%_ > 250 °C) and a clear increase in glass transition temperatures with decreasing β-myrcene content in the polymeric shell. PM_m_-PS_n_ cross-linked copolymer shells also proved to be good protectors for the [EMIM][DCA] core, where PM-IL had the greatest IL content with 54 wt % followed by 46, 31, 28, and 26 wt % for PM_7_-PS_3_, PM_5_-PS_5_, PM_3_-PS_7_, and PS-IL, respectively. Increasing β-myrcene content in the copolymer shell led to greater encapsulated ionic liquid, which resulted in higher CO_2_ absorption capacity. Also, styrene content plays an important role in preventing the hydrocarbon polymer chains of poly(β-myrcene) from packing into close arrangements leading to self-assembly toward inducing the formation of a porous shell morphology. Increased porosity led to an increased rate of mass transfer-controlled CO_2_ sorption by increasing the surface contact area and access to the encapsulated ionic liquid. Among the PM_m_-PS_n_ microparticles formed, the IL microcapsule sample with 50/50 weight ratio of β-myrcene/styrene showed the best synergistic properties with an ionic liquid encapsulation efficiency of 31 wt %, spherical particle formation (perimeter of 32.2 μm), relatively large average pore sizes of 0.75 μm, high CO_2_ sorption capacity of 0.5 mmol CO_2_/g sample, and a short saturation absorption time after 20 min. Therefore, this work provides a facile approach to improving the mass transfer rate of CO_2_ absorbing ionic liquids by encapsulating the ionic liquid within a hydrophobic, copolymer shell.

## Supporting Information Available

Experimental details for preparing myrcene/styrene cross-linked copolymer microparticles, FTIR spectra of [EMIM][DCA]-encapsulated microparticles and the bulk ionic liquid, FTIR spectra showing complete removal of the ionic liquid after successive acetone washes, DSC data of [EMIM][DCA], photographs of the studied microcapsules suspended in *n*-heptane before and after being subjected to a high centrifugal force, FE-SEM images of prepared microparticles, and nitrogen absorption data for IL-encapsulated microparticles (PDF)
