# Supplementary material for: Structure–Property Relationships of CO2 Absorbing Core–Shell Microparticles with Encapsulated Ionic Liquid
Source: ACS Omega. 2023 Jun 17;8(26):24032–41. doi: 10.1021/acsomega.3c02975 (PMC10324060; doi:10.1021/acsomega.3c02975)
Supplement: Supplementary file 1 — ao3c02975_si_001.pdf [file ao3c02975_si_001.pdf]

Supporting Information

# Structure-Property Relationships of $CO_2$ Absorbing Core-Shell Microparticles with Encapsulated Ionic Liquid

*Ai-Nhan Au-Duong, Asem Abdulahad**

[1] Department of Chemistry, Xavier University of Louisiana, New Orleans, Louisiana, USA.

*Author to whom all correspondence should be addressed

Correspondence to:

*Asem Abdulahad aabdulah@xula.edu (ORCID: )

Keywords: $CO_2$ capture, core-shell microparticle, ionic liquid, encapsulation, macroporous shell, poly(myrcene-co-styrene)

**Table S1.** Recipe of emulsion mixture for synthesis of microparticles.

| Sample | β-myrcene (g) | Styrene (g) | HD (g) | EMIM:DCA (g) | DI-W (g) | Heptane(g) | Tween 20 (g) | Span 80 (g) | Reaction time (h) |
|---|---|---|---|---|---|---|---|---|---|
| **PM** | 2.83 | 0 | 1.2 | 0 | 2.2 | 14 | 0.056 | 0.4 | 24 |
| **PM-ILs** | 2.83 | 0 | 1.2 | 1.2 | 1 | 14 | 0.056 | 0.4 | 24 |
| **$PM_7$-$PS_3$** | 1.98 | 0.64 | 1.2 | 0 | 2.2 | 14 | 0.056 | 0.4 | 24 |
| **$PM_7$-$PS_3$-ILs** | 1.98 | 0.64 | 1.2 | 1.2 | 1 | 14 | 0.056 | 0.4 | 24 |
| **$PM_5$-$PS_5$** | 1.42 | 1.07 | 1.2 | 0 | 2.2 | 14 | 0.056 | 0.4 | 24 |
| **$PM_5$-$PS_5$-ILs** | 1.42 | 1.07 | 1.2 | 1.2 | 1 | 14 | 0.056 | 0.4 | 24 |
| **$PM_3$-$PS_7$** | 0.85 | 1.5 | 1.2 | 0 | 2.2 | 14 | 0.056 | 0.4 | 8 |
| **$PM_3$-$PS_7$-ILs** | 0.85 | 1.5 | 1.2 | 1.2 | 1 | 14 | 0.056 | 0.4 | 8 |
| **PS** | 0 | 2.14 | 1.2 | 0 | 2.2 | 14 | 0.056 | 0.4 | 8 |
| **PS-ILs** | 0 | 2.14 | 1.2 | 1.2 | 1 | 14 | 0.056 | 0.4 | 8 |

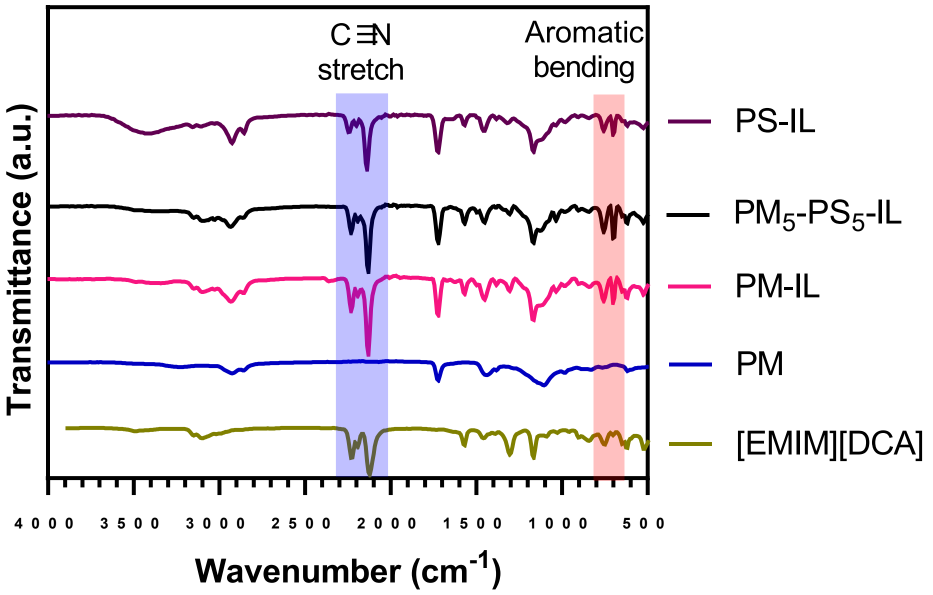


**Figure S1.** ATR-FTIR spectra of the studied microparticles.

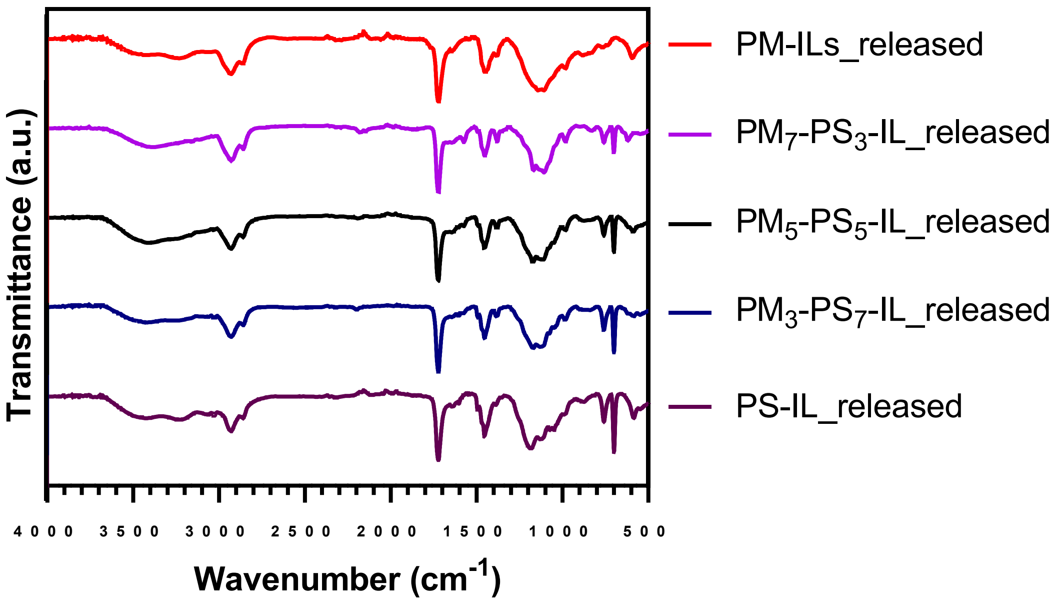


**Figure S2.** FTIR-ATR spectra of ILs after removing [EMIM][DCA] from the $PM_m$-$PS_n$ microcapsules.

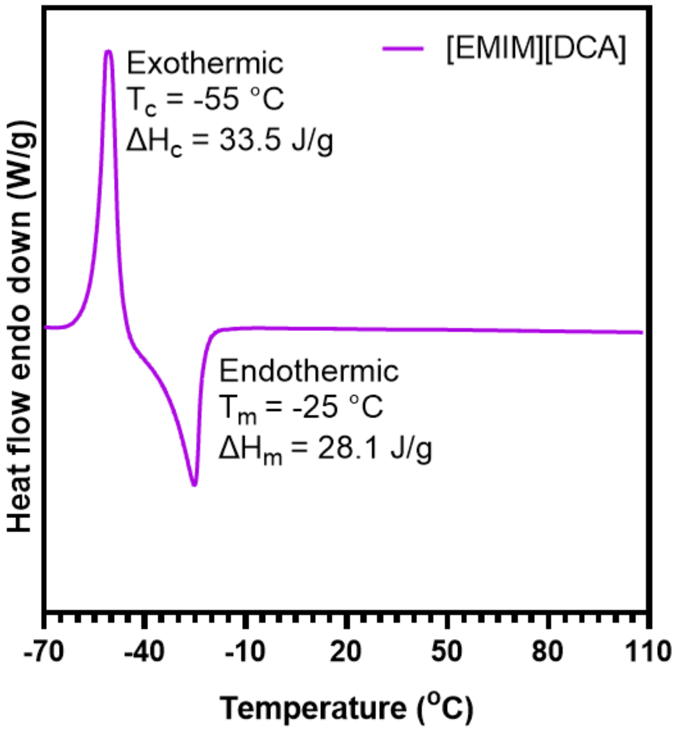


**Figure S3.** Differential scanning calorimetry of [EMIM][DCA] showing both exothermic crystallization and endothermic melting transitions.

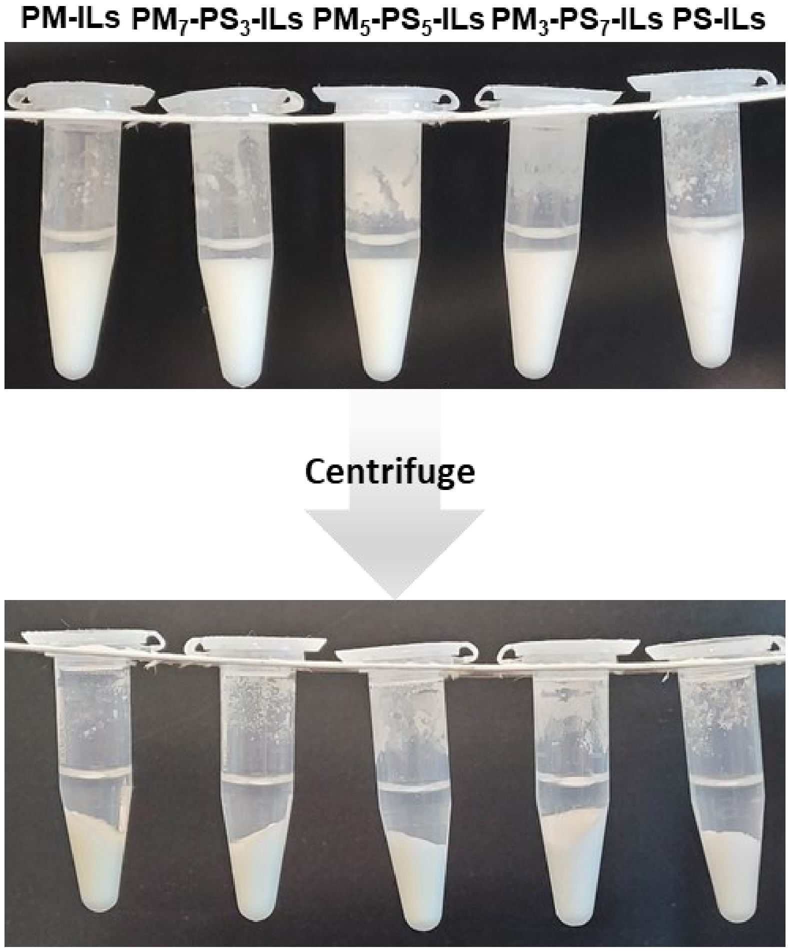


**Figure S4.** (a) Suspending the microcapsules in n-heptane and (b) centrifuging these suspensions from 3000 rpm to a high centrifugal force of 8000 rpm and a sequential increment of 500 rpm in centrifugation speed per minute for each centrifuging step.

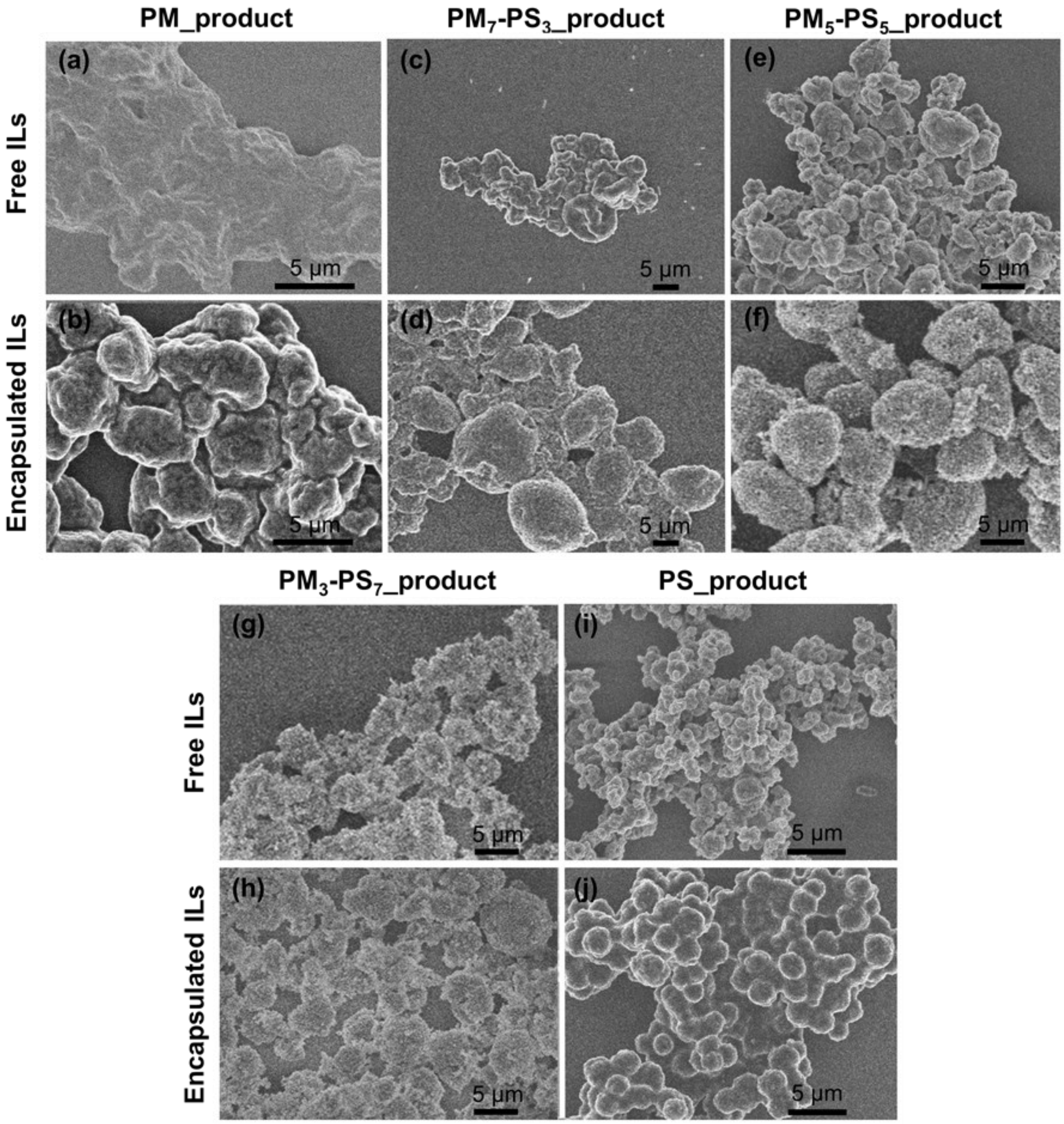


**Figure S5.** Observation for the morphology by FE-SEM images of (a) PM, (b) PM-ILs, (c) $PM_7$-$PS_3$, (d) $PM_7$-$PS_3$-ILs, (e) $PM_5$-$PS_5$, (f) $PM_5$-$PS_5$-ILs, (g) $PM_3$-$PS_7$, (h) $PM_3$-$PS_7$-ILs, (i) PS, and (j) PS-ILs.

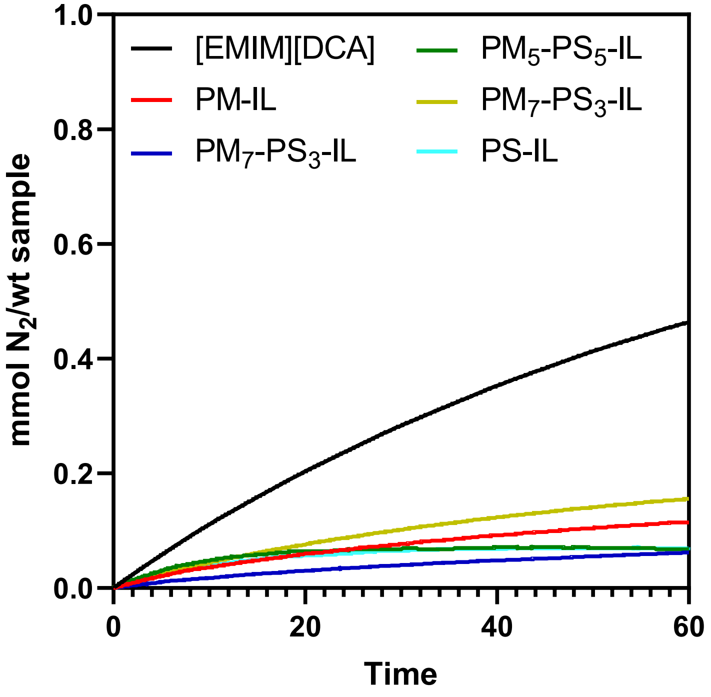


**Figure S6.** Nitrogen absorption of ILs-encapsulated microparticles expressed per mass of prepared samples.
